# Folate intake and the risk of endometrial cancer: A meta-analysis

**DOI:** 10.18632/oncotarget.13211

**Published:** 2016-11-08

**Authors:** Li Du, Yulong Wang, Hang Zhang, Hong Zhang, Ying Gao

**Affiliations:** ^1^ State Key Laboratory of Genetic Engineering and Institute of Biostatistics, School of Life Sciences, Fudan University, Shanghai, China; ^2^ Key Laboratory of Nutrition and Metabolism, Institute for Nutritional Sciences, Shanghai Institutes for Biological Sciences, Chinese Academy of Sciences, Shanghai, China

**Keywords:** folate, intake, endometrial cancer, risk, meta-analysis

## Abstract

**Background:**

Folate may involve in various aspects of carcinogenesis. However, the relationship between folate intake and risk of many cancers, including endometrial cancer, is still inconclusive. We conducted a meta-analysis to systematically review the association.

**Methods:**

Relevant studies were searched through three electronic databases (PubMed, Embase, and Web of Science) up to April 4, 2016. Population based prospective or case-control studies involving in investigating folate intake and risk of endometrial cancer were considered as eligible. Three investigators independently extracted data. Controversies were reconciled by discussing with a fourth investigator. Effect sizes of studies were pooled via a random effects model. Thereafter to explore the origin of heterogeneity among results of studies, a mixed effects model was employed with study design and dose of folate intake taken as covariates.

**Results:**

Nine case-control studies and five cohort studies were included in the current meta-analysis. The result pooled from the highest category suggested a marginal negative association between folate intake and risk of endometrial cancer (OR=0.89 95% CI: 0.76-1.05). Based on the mixed effects model, in the highest category, the risk showed an increasing trend along with increment of folate intake (5% risk increase per 100μg/d, P=0.01).

**Conclusion:**

A marginally negative association was observed between folate intake and endometrial cancer, which might subject to a threshold effect. More finely designed perspective studies or randomized trials are still needed to confirm the association.

## INTRODUCTION

As a cofactor of the de novo synthesis of purine and thymidylate and the main supplier of one carbon unite, folate plays a critical role in keeping genetic and epigenetic stability of DNA [1]. The deficiency of folate will lead to a serial of abnormality, including breaks in the DNA strand, enhanced mutation rates, impairs in DNA repair mechanism and alternations of methylation status in genome scale [2].

Animal experiments and epidemiological studies had shown a link between increased risk of various cancer and folate deficiency [3–5]. However, folate may also motivate cell proliferation, which makes the relationship between folate intake and cancer risk more complicated [6, 7].

Some epidemiological studies suggested another story about the relationship of folate intake and cancer risk. A nested case-control study conducted in Sweden based on 226 cases and 437 matched controls suggested that the relationship between plasma folate concentration and the risk of colorectal cancer was in a bell-shaped manner [8]. Some other studies reported a positive association between folate and risk of cancers, such as breast cancer (odds ratio, OR: 1.19, 95% CI: 1.01, 1.41) [9], prostate cancer (OR: 2.0; 95% CI: 1.1, 3.7) [10], and ovarian cancer (relative risk, RR: 1.21; 95% CI: 0.94, 1.63) [11]. Briefly, doubts about the role folate playing in carcinogenesis are emerging, at least for certain types of cancer, based on certain populations.

Being one of the most common gynecologic malignancy tumor, the endometrial cancer develops in over 200,000 women worldwide, deprives above 42,000 lives every year [12]. The relationship between folate intake and risk of endometrial cancer is still inconclusive. The OR's of studies range from 0.57 to 1.71, with only few ones have statistical significant associations [13–26]. Therefore, we aimed to evaluate the association between endometrial cancer risk and folate intake, and explore the source of heterogeneity among studies, through systematic reviewing published articles.

## RESULTS

### Search results

**Figure 1:**
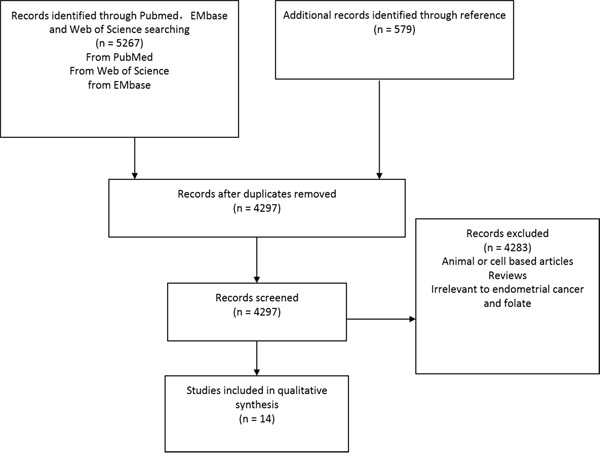
Identification process for eligible studies

The search process was presented in Figure 1. From PubMed, EMbase, and Web of Science with key words of “folate” paired with “cancer” and “endometrial”, 5267 articles were screened. From references of articles and relevant reviews 579 more articles were identified. There were 1536 duplicates were removed and left 4297 articles. Among them, 4283 were excluded because they were based on animal or cell lines, or irrelevant. Fourteen studies were finally included in the current meta-analysis [13–26].

### Description of the included studies

All studies identified were human based epidemiological studies focusing on folate intake and risk of endometrial cancer, with participants of post-menopause women. Five of 14 studies were perspective cohort studies. All five cohort studies provided dietary data from follow-up. The other nine were retrospective case-control studies, with four based on hospital and five based on population. The years of follow-up for cohort studies and duration of data collection after diagnosis for case-control studies were listed in Table 1. Eleven studies were conducted in Canada and USA, the other three were conducted in China, Mexico, and Italy and Switzerland respectively. The intake of folate was all quantified by food frequent questionnaire. Though based on the same population, the study of Uccella et al [20] reported the OR of two different subtypes of endometrial cancer separately, thus both of them were adopted. Detailed characteristics of studies were listed in Table 1.

**Table 1:** Characteristics of studies

References	Year	Geographic regions	Case-controls or cohort size	Age	Food frequency questionnaire (items)	Folate intake calculation method	Time frame*	Covariates	Exclusion of hysterectomy
**Hospital-based case-control**									
**Tavani et al [19]**	2012	Italy& Switzerland	454/908	61.5^#^	78	IFCD	10 years	Age.Alc.B. Edu.Engy.R.S	Not mention
**Yeh et al [22]**	2009	USA	541/541	27-96	44	USDA	16 years	Age.B.S. Engy.H.R	Yes
**Martinez et al [17]**	2005	Mexico	85/629	18-81	116	Not mention	2 years	Age.Alc.B. Engy.H.R	Yes
**McCann et al [16]**	2000	USA	232/ 639	40-85	172	USDA	5 years	Age.Alc.B. Engy.H.R.S	Yes
**Population-based case–control**									
**Biel et al [21]**	2011	Canada	506/ 981	30-79	124	USDA^@^	4 years	(Age).Alc.B. Engy.H.S.R	Not mention
**Xu et al [25]**	2007	China	1204/ 1212	30-69	71	USDA& CDA	6 years	Age.Alc.B.Edu.Engy.H.R	Yes
**Paynter et al [18]**	2004	USA	201/ 603	30-55	61	USDA	16 years	(Age).Alc. Engy	Not mention
**Jain et al [23]**	2000	Canada	552/ 562	40-59	142	USDA	5 years	Age.Alc.B. Engy.H.R.S	Yes
**Potischman et al [15]**	1993	USA	399/ 296	20-74	60	Not mention	5 years	(Age).Alc.B. (Engy).H.R.S	Yes
**Cohort Studies**									
**Liu et al ^¶^[13]**	2013	USA	788/ 121700	30-55	Not mention	USDA	26 years	Age.B.H.R.S	Yes
**Uccella et al ^¶^[20]**	2011	USA	471/ 23356	55-69	126	USDA	17 years	Age.Alc.B. Engy.H.R.S	Yes
**Uccella et al ^®^[20]**	2011	USA	71/ 23356	55-69	126	USDA	17 years	Age.Alc.B. Engy.H.R.S	
**Kabat et al [24]**	2008	Canada	426/ 34748	40-59	86	USDA	16.4 years (average)	Age.Alc.B.Edu.Engy.H.R.S	Yes
**Jain et al [14]**	2000	Canada	221/ 56837	30-79	86	USDA	10.3 years (median)	Age.B.Edu.Engy.H.R.S	Not mention

* Years of follow-up for cohort studies and duration of data collection after diagnosis for case-control studies.

# Median age.

¶ Focusing only on type I endometrial cancer.

® Focusing only on type II endometrial cancer.

@ When estimating total folate intake, the amount of synthetic folic acid was multiplied by 1.7 in order to account for the greater bioavailability of synthetic folic acid.

Folate intake calculation method: IFCD=Italian food composition database, USDA=composition database from U.S Department of Agriculture, CDA=composition database from China Department of Agriculture

Covariates: Age=Age, Alc=Alcohol, B=BMI/weight, Edu=Education, Engy=Total Energy, H=HRT/ERT use, R=Reproductive factors, S=Smoking. (Age): matched on age. (Engy) Energy from carbohydrate calories.

### Data quality

Potential confounding factors including age, total energy intake, alcohol intake, and BMI were controlled or matched while estimating ORs or RRs. Twelve out of 14 studies each had a sample size of more than 200 cases [12-16, 18-25]. The sample size of the other two articles were 85 and 71 cases respective [17, 20]. Thirteen of 14 studies calculated the intake of folate directly from food frequent questionnaires. When estimating total folate intake, Biel et al [21] multiplied the amount of synthetic folic acid by 1.7 before it was added to folate from food in order to account for the greater bioavailability of synthetic folic acid.

### Association between folate intake and risk of endometrial cancer

**Figure 2:**
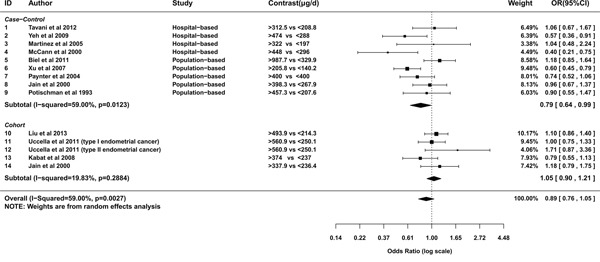
Meta-analysis of subgroup by design of studies

**Figure 3:**
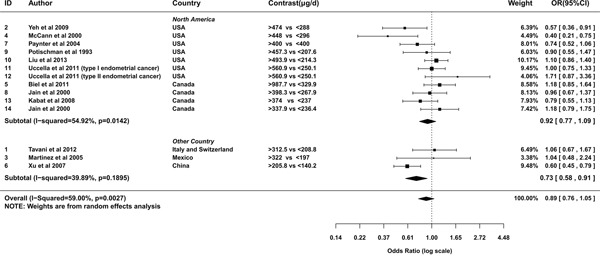
Meta-analysis of subgroup by geographic regions of studies

A random effects model was used to pool the ORs of highest category versus the lowest (*I*^2^=59%, *P*heterogeneity=0.003). The overall result suggested a marginal negative relationship between folate intake and the risk of endometrial cancer (OR= 0.89; 95% CI= 0.76-1.05; Figure 2). The OR pooled from case-control studies was statistically significant (OR= 0.79; 95% CI= 0.64-0.99), evidently apart from that summarized from cohort studies (OR= 1.05; 95% CI= 0.9-1.21; Figure 2). From studies conducted in North America, the relationship was neutral (OR= 0.92; 95% CI= 0.77-1.09; Figure 3). Whereas studies conducted outside North America showed a significantly negative relationship (OR= 0.73; 95% CI= 0.58-0.91; Figure 3). To assess the robustness of the relationship, we conducted a sensitivity analysis. If the weight of Xu et al [25] was decreased from 66.3% to 51.66%, alias, the difference of weights between Xu et al and summation of the other two studies decreased 29.28%, the association would alter to marginal significant(OR= 0.78; 95%CI=0.62-1). If the weights of three studies were set equal, the estimation of effect size was still negative (OR=0.87). However, it was not statistically significant (95%CI=0.63-1.18).

To explore the origin of heterogeneity among study results, in the highest category, we employed a mixed effects model with study design and dose of folate intake as covariates. The risk showed a significant increasing trend along with further increment of folate intake (5% risk increase when the folate intake increases 100μg/d, P=0.01). Meanwhile comparing to cohort studies, the mean lnOR of case-control studies was significantly lower (β = −0.249, P=0.002).

We also applied the mixed effects model to the second highest category. No significant publication bias was observed (Begg's test P=0.367; Egger's test P=0.541). The pattern of results was similar as that in the highest category, with a comparable increasing trend (6% risk increase per 100μg/d, P=0.057) and a more substantial difference of mean lnORs between different study designs (β = −0.3666, P<0.001).

When it comes to the third higher category, The P values for Begg's test and Egger's test were 0.638 and 0.22 respectively, suggested no significant publication bias. Because of the insignificant heterogeneity (*I*^2^=30.15%, *P*heterogeneity=0.15), additional covariates were not necessary. Interestingly, if we incorporated the two covariates into the mixed effects model in the same way as the two higher categories, the trend of risk relation to intake of folate turned neutral (1% risk decrease per 100μg/d, P=0.899); yet the effect of study design remained relative constant, with an estimation of -0.21 and a P value of 0.046 for the difference of mean lnOR between two subgroups.

### Publication bias

**Figure 4:**
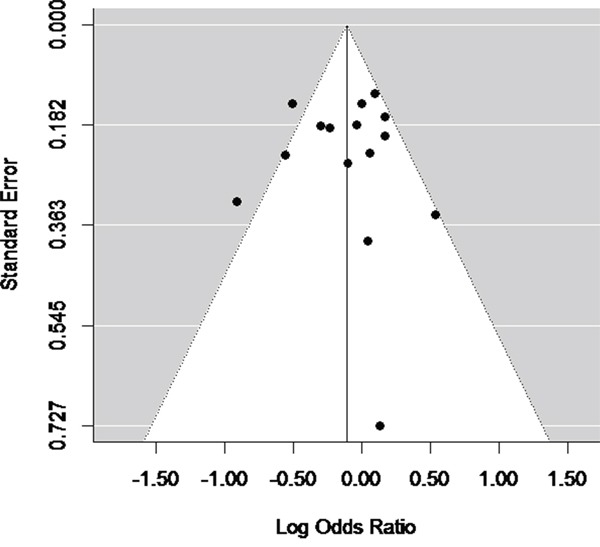
Funnel plot (with pseudo 95% CIs) of all included studies

Egger's test and Begg's test was performed in all 14 studies. Funnel plots were used for graphical representation. Significant publication bias was not observed, as suggested by Begg's test and Egger's test (*P_Begg_*=0.67), *P_Egger_*=0.37, Figure 4).

## DISCUSSION

To explore the association between folate intake and the risk of endometrial cancer, we conducted this meta-analysis by including 14 independent studies. In the highest category, summarized by a random effects model, the folate intake showed a marginal negative association with the risk of endometrial cancer. Through a stratified analysis, a significant negative association was observed in studies conducted outside North America and case-control studies. Then we further explored the heterogeneity through a mixed effects model, taking dose of folate intake and study design as covariates. The model was applied to all three higher categories. The difference of mean lnOR between case-control studies and cohort studies kept numerically relative stable and statistically significant among all three categories. A positive trend between folate intake and endometrial cancer risk was observed in the two higher categories.

Folate is extensively involved in various kinds of biological processes. The impact of folate upon risk of endometrial cancer may be blurred by different roles folate plays. As an important cofactor in DNA synthesis and one carbon unit metabolism, folate may help to inhibit carcinogenesis through promoting DNA repair, decreasing DNA mutagenesis and preventing aberrant DNA methylation [27]. Observational studies and clinical trials about various kinds of cancer supported the negative relationship of folate intake and cancer [3-5, 28].

However, on other side, excess folate may also facilitate DNA synthesis in rapid proliferating cells, promote the growth and progression of already existed neoplasma [29]. Which is the rationale of anti-folate drug implementation in cancer therapy [2]. In addition, according to Troen et al [30], dietary folate intake, above a certain intake level, may enervate the cytotoxity of natural killer (NK) cell, an important part of nonspecific immune response system. Women in the lower tertile with folate intake <233μg/d and a supplement up to 400μg/d had better immune function than those in the same tertile without supplement. However, for those had a dietary folate intake more than 233μg/d, an additional supplement of more than 400μg/d would lead to impairments of the NK cell function [30].

Some study design related factors could affect the outcomes of studies and have impact on the pooled OR. First, comparing to the estrogen dependent Type I, type II endometrial cancer is often related to p53 mutations, which are associated with DNA damage and abnormal cell proliferation. Therefore patients may be more susceptive to folate intake alternations [20]. However, the majority of studies included in our meta-analysis failed to distinguish two subtypes for patients, which increased the heterogeneity among subjects and decreased the power of detecting difference. Besides, the proportion of two subtypes may be different in different populations. Thus it may also increase heterogeneity of outcomes among studies. Second, folate from natural food and fortification had different effects in 13 of 14 studies. Folate is mainly fortified in the form of folic acid, a more stable analogue with significant higher bioavailability [34]. In North America, the average folic acid intake is about 400μg/d [32], approximately twice the amount of folate from natural food. Thus the indiscrimination the two source of folate would lead to an underestimation of folate intake and a deviation in the estimation of effect size, especially for 9 studies conducted after the initiation of mandatory fortification in North America. Therefore, to confirm the association between folate intake and risk of endometrial cancer, studies being able to control the modification effect of more factors, including but not limited to those mentioned above, are still urgently needed.

The counterbalancing of folate may also lead to a threshold effect, which might be helpful to explain the positive trend of risk along with further increase of folate intake. Song et al [31] observed a dose-response positive relationship of folate supplement and risk of colorectal cancer in mice. To address the effect of large increase of folate intake [median (95% CI) of plasma folate concentration: from 12.5 (11.8, 12.9) in NHANES to 32.2 (30.1, 33.8) nmol/L in NHANES III] on colorectal cancer after the fortification [32], Mason et al collected the incidence rate of two independent populations from USA and Canada respectively [33]. Both populations experienced a persistent decreasing of incident ratio in decades until mid-1990s. However, the downward trend reversed abruptly thereafter. For USA it climaxed in 1998, for Canada in 2000, and kept at an incidence of 40 to 60 per million higher than that of 1996. By contrast, in this meta-analysis, the positive trend could not be observed in the 3^rd^ highest category, was marginally significant in the 2^nd^ highest category, became significant in the highest category.

In the current study, a negative association between folate intake and risk of endometrial cancer was observed in case-control studies. Since the difference of mean lnOR between case-control studies and cohort studies kept significant and relative stable among all three categories, recall bias inherited in the retrospective study might contribute to that deviation. When patients recalling their dietary, they tended to underestimation of folate intake and overestimation of protective effect of folate, especially for five population-based case-control studies.

Interestingly, a negative association was also observed in studies conducted outside North America. The sensitive analysis showed that the association was with moderate robustness. However, till now, through a comprehensive literature search there are still only three eligible studies identified. What's more, all three studies are retrospective study, thus they may also subject to the recall bias. Which weakened the solidity of conclusion, and is a major limitation of this meta-analysis.

This meta-analysis has some advantages. The mixed effects model enabled us to evaluate the effect size of covariates separately. By incorporating dose of folate intake into model, we could explore variation trend of the association. By taking study design as covariates, we could control the effects of study design, which is always a primary concern.

In conclusion, our meta-analysis suggested a marginal negative relationship between endometrial cancer risk and folate intake. The effect of folate may have a threshold effect. To illuminate the relationship of folate and endometrial cancer, more finely designed cohort and randomized clinical studies are needed. Moreover, subtype of endometrial cancer and source of folate need to be considered.

## MATERIALS AND METHODS

### Literature search

The process of the meta-analysis in this article was complied with the Preferred Reporting Items for Systematic Reviews and Meta-Analysis Statement (PRISMA) issued in 2009 [35]. We systematically searched all human studies about folate intake and the risk of endometrial cancer up to August 31, 2016 from PubMed (http://www.ncbi.nlm.nih.gov/pubmed), EMbase (http://www.embase.com), and Web of Science (http://www.webofknowledge.com). The references of studies and relevant reviews were also checked.

The key words for searching were as following: (“folate” OR “folic” OR “folacin”) OR (“pteroyl-l-glutamic acid” OR “pteroyl-l-glutamate” OR “pteroylmonoglutamic acid”)) paired with ((“endometrial neoplasm”) OR ((“malign” OR “cancer” OR “carcinoma” OR “tumor”) AND (“endometrial” OR “corpus uterine” OR “uterine”)).

The inclusion criteria for studies are as following:

- The research was a human based case-control study, prospective study or clinical trial.
- The interests of article included investigating the association between folate intake and risk of endometrial cancer.
- The effect size (hazard ratio [HR], odds ratio [OR], or risk ratio [RR]) and their confidence intervals (CIs) were available.

The exclusion criteria were as following:

- Studies based on cell lines or animal.
- The article is not written with English.
- Reviews, case-report, editorials or commentaries.

Three investigators (Li Du, Yulong Wang and Hang Zhang) identified eligible articles independently according to the aforementioned criteria. Controversies on article inclusion among the three reviewers were resolved through discussing with a fourth investigator (Ying Gao).

### Data extraction and quality assessment

Data on the study design, country, sample size, age, food frequency questionnaire items, time frame, exclusion of hysterectomy together with folate intake level of each category, effect size (OR for case control study, RR for cohort study), and 95% CI were collected. If crude OR or RR was reported together with multivariable adjusted-effect estimates, we chose the estimation that had been fully adjusted for the potential confounders.

The quality of studies was accessed by sample size and whether important confounders such as alcohol intake, total energy intake, body mass index and age had been adjustment for.

### Statistical analysis

All statistical analyses were performed with R, version 3.1.2 (Package metafor). The primary outcome was defined as odds ratio (OR). Thus risk ratios (RR) together with their variance were converted to lnORs for further analysis [26]. Heterogeneity across studies was accessed according to the Cochrane *Q* statistic and *I^2^* statistic. As long as the P value for heterogeneity was smaller than 0.05 or the *I^2^* statistic was greater than 25%, results of studies were considered as significantly heterogeneous and a DerSimonian and Laird random effects model was used to pool the results [36, 37]. Otherwise the classical fixed effect model was employed. Modification effects of study design and countries (with or without mandatory folic acid fortification) were explored by stratified analysis. Countries with mandatory folic acid fortification include Canada and USA. Due to the limited number of studies conducted outside North America, to assess the robustness of the relationship, a sensitivity analysis was conducted by adjusting the weights of studies.

To explore the origin of heterogeneity among studies, a mixed effects model was applied to three higher categories as most of candidate studies were in quartile. Since the mandatory folic fortification in North America had led to a substantial difference in folate intake of populations in different country, the dose of folate intake, as well as study design were chosen as covariates of model [38, 39]. Within every study, the mean folate intake in each category was estimated by method proposed by Chene and Thompson [40].

If the population on which a study based was divided into less than four categories, for instance, in tertiles, the two lnORs were allocated for pooling results of the highest and the second highest category. At the second highest category, study of Randi et al [18] was excluded because the population was divided into two quantiles. Then at the third highest category, the research of Martinez et al [17] with only 3 categories was also excluded.

The results of every study in all the models above were negative variance weighted. Publication bias was detected by Egger's test and Begg's test, then graphically represented by funnel plots.
